# Bioassays for Identifying and Characterizing Plant Regulatory Peptides

**DOI:** 10.3390/biom13121795

**Published:** 2023-12-14

**Authors:** Alexander Skripnikov

**Affiliations:** 1 Shemyakin—Ovchinnikov Institute of Bioorganic Chemistry, Miklukho-Maklaya St. 16/10, 119997 Moscow, Russia; deptbioorg@gmail.com; 2 Department of Biology, Lomonosov Moscow State University, 119234 Moscow, Russia

**Keywords:** plant peptides, bioassay, mass spectrometry, putative peptides, regulatory peptides, root growth assay, peptide signaling

## Abstract

Plant peptides are a new frontier in plant biology, owing to their key regulatory roles in plant growth, development, and stress responses. Synthetic peptides are promising biological agents that can be used to improve crop growth and protection in an environmentally sustainable manner. Plant regulatory peptides identified in pioneering research, including systemin, PSK, HypSys, RALPH, *At*Pep1, CLV3, TDIF, CLE, and RGF/GLV/CLEL, hold promise for crop improvement as potent regulators of plant growth and defense. Mass spectrometry and bioinformatics are greatly facilitating the discovery and identification of new plant peptides. The biological functions of most novel plant peptides remain to be elucidated. Bioassays are an essential part in studying the biological activity of identified and putative plant peptides. Root growth assays and cultivated plant cell cultures are widely used to evaluate the regulatory potential of plant peptides during growth, differentiation, and stress reactions. These bioassays can be used as universal approaches for screening peptides from different plant species. Development of high-throughput bioassays can facilitate the screening of large numbers of identified and putative plant peptides, which have recently been discovered but remain uncharacterized for biological activity.

## 1. Introduction

Over the past three decades, peptides have taken an important place among the key regulators of plant growth, morphogenesis, and stress and defense reactions. Traditionally, peptides are defined as short chains of amino acids, typically containing no more than 50 residues, with a molecular weight ranging from 5 to 6 kDa [1]. However, some researchers extend the peptide classification to include amino acid polymers with up to 100 residues [2]. In contrast to the regulatory peptides of animals and humans, most of the regulatory peptides of plants have been discovered in recent years [2,3]. The study of new plant regulatory peptides is of great fundamental and applied significance because they can also be used as low-molecular-weight bioregulators in agriculture. Plant peptides play an important role in regulating various aspects of plant growth and development, stress resistance, and defense reactions.

Plant regulatory peptides predominantly serve as ligands for membrane receptors, the major group of which are receptor-like kinases (RLKs). Plant RLKs have an intracellular kinase domain, a single membrane domain, and extracellular domain. Plant RLKs are similar to animal receptor tyrosine kinases (RTKs) in domain structure, but they have several key differences [4,5]. Plant RLKs binding peptide ligands have extracellular leucine-rich repeat (LRR) domains, which are thought to play a role in protein–protein interactions and peptide binding. RTKs have a variety of different extracellular domains, depending on the specific RTK. One of the largest group of plant RLKs have serine/threonine kinase domains, while RTKs have tyrosine kinase domains. Differences in kinase domains may lead to activation of different signaling pathways. 

The exploration of plant regulatory peptides commenced in 1991 with the identification of systemin, an 18-amino acid peptide that orchestrates systemic acquired resistance in *Solanaceae* plants [6]. The discovery of systemin influenced the initiation of studies into the search and identification of new plant regulatory peptides [3]. Novel plant peptides have been identified through the purification of peptide fractions from plant extracts, with mass spectrometry playing a crucial role in elucidating their amino acid sequences. Bioassays employing *in vitro* cultures of cells and plant seedlings have proven instrumental in elucidating the functions of novel plant peptides. Several bioassays originally employed for characterizing early-discovered plant regulatory peptides have evolved into versatile *in vitro* test systems for investigating both newly isolated and putative plant peptides. Remarkably, the early-discovered plant regulatory peptides continue to be actively investigated and hold substantial applied potential. Prominent examples include cell proliferation regulators like the 5-amino acid PSK (phytosulfokine) [7,8] and the 18-amino acid peptide PSY1 (Plant peptide containing sulfated tyrosine 1) [9,10]; defense peptides like systemin [11,12,13,14,15,16,17], the 18-amino acid HypSys (hydroxyproline-rich systemin) [18,19], *At*Pep1, the 23-amino acid peptide isolated from extracts of *Arabidopsis* leaves, exhibiting characteristics of an endogenous elicitor of the innate immune response [20,21,22,23,24,25,26], and the 49-amino acid RALF peptide (Rapid alkalinization factor) [27,28]; apical meristem regulator, the 12-amino acid peptide CLV3 (CLAVATA3) [29,30,31,32], and other members of the CLE (CLAVATA3/EMBRYO SURROUNDING REGION-related) [30] peptide family such as the 12-amino acid vascular stem cell differentiation factors TDIF and CLE41/44 [33,34] and root growth regulator CEP1 [34,35] (Table 1).

The biological activity of hundreds of newly isolated and putative plant peptides remains uncharacterized. Despite the explosive growth in publications on plant peptides, the number of isolated and characterized regulatory peptides is rather modest. In general, the number of plant regulatory peptides with verified structure currently amounts to several dozen, although the bioinformatic search of potential precursors of the secreted peptides indicates that it could potentially be hundreds of regulatory peptides [2,3,32,49]. The isolation, identification, and characterization of new regulatory peptides remains a highly challenging task due to their extremely low concentrations in plant cells and tissues [49]. State-of-the-art nano-LC-MS/MS and bioinformatics have enabled the identification of hundreds of peptides in plant extracts [50,51], but the biological functions of most of them remain unknown. This brief review will describe the role of bioassays in characterizing new plant peptides.

## 2. Mass Spectrometry Is a Powerful Tool for the Identification of Plant Peptides

**Figure 1:**
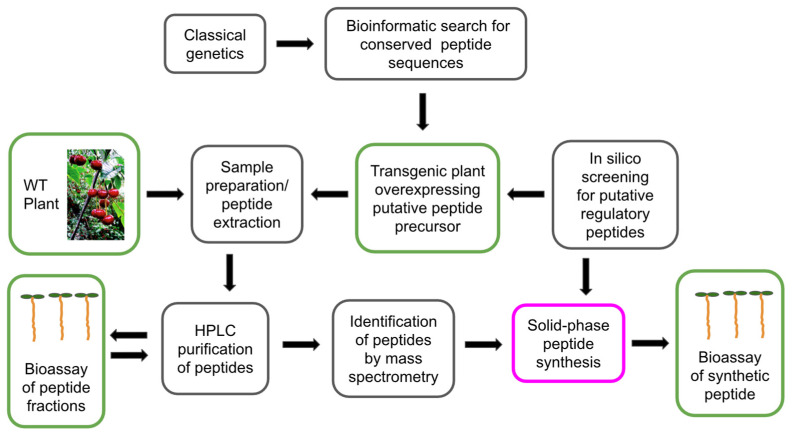
Strategies and workflow for the search and characterization of new regulatory plant peptides. Bioassays are used as analytical tools for bioassay-guided peptide purification and for evaluating the biological activity of synthetic peptides.

**Figure 2:**
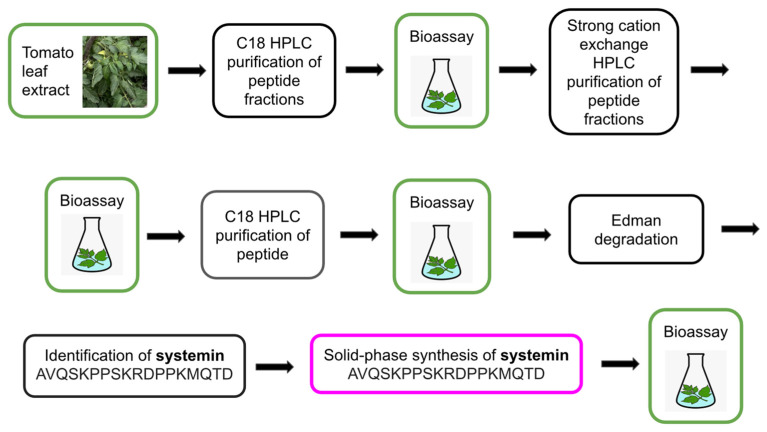
Bioassay-guided isolation and identification of the 18-amino acid peptide systemin from tomato leaves. Bioassays were conducted by treating tomato seedlings with the peptide fractions or synthetic 18-amino acid peptide systemin and measuring the accumulation of the proteinase inhibitor in tomato cuttings using the immunoassay [6].

**Figure 3:**
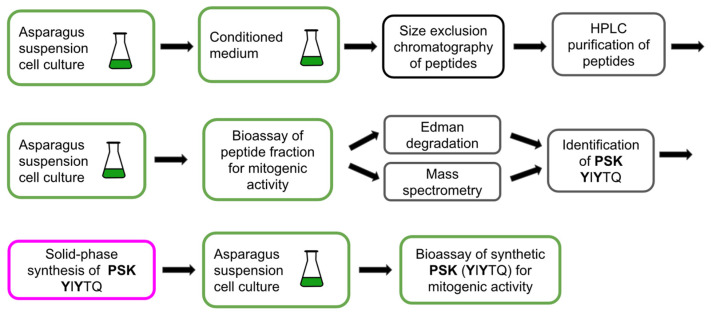
Isolation and identification of the peptide phytosulfokine (PSK) from asparagus cells. Asparagus cells were used as the source of peptide preparation and as a unicellular mitogenic model for bioassays of both peptide fractions and synthetic peptide PSK. The structure of phytosulfokine was determined by Edman degradation and mass spectrometry [7]. Two tyrosine residues are sulfated (shown as bold).

**Figure 4:**
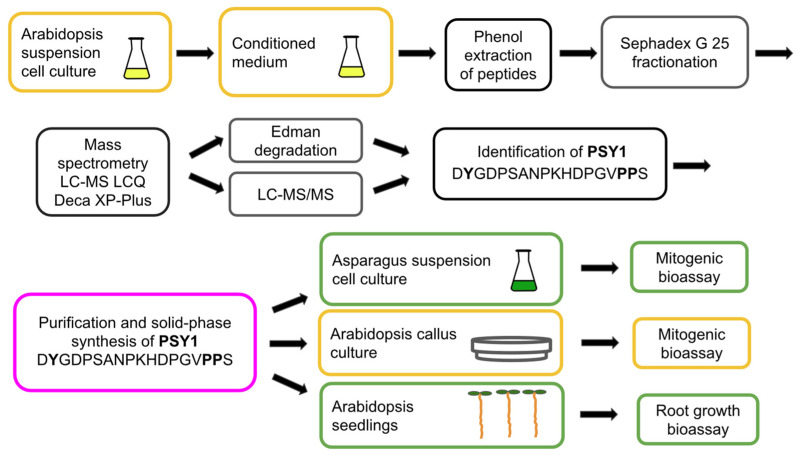
Isolation and identification of the peptide PSY1. The peptide fraction was extracted using phenol from the conditioned medium of *Arabidopsis* suspension cell culture. The structure of PSY1 was established using Edman degradation and LC-MS/MS mass spectrometry. The 18-amino acid peptide PSY1 contains one sulfated tyrosine and two hydroxylated proline residues (bold). PSY1 was assayed for mitogenic activity in suspension-cultivated asparagus and *Arabidopsis* cells, as well as in *Arabidopsis* seedlings using a root growth assay [9].

**Figure 5:**
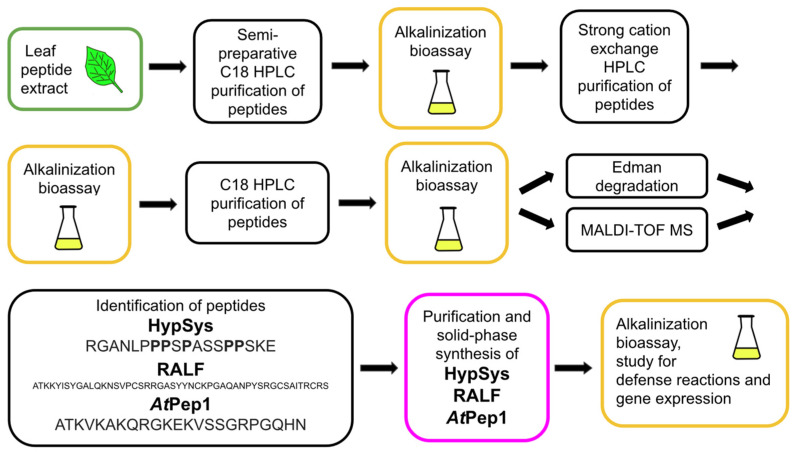
Bioassay-guided isolation and identification of the peptides HypSys and RALF from tobacco leaf extract and peptide *At*Pep1 from *Arabidopsis*. Peptide fractions and the synthetic peptides were investigated using the suspension culture of *Nicotiana tabacum* cells as a bioassay system to detect rapid liquid medium alkalinization accompanying stress signal [18]. The 18-amino acid peptides HypSys and 5 kDa RALF peptide containing four cysteine residues were isolated and assayed for biological activity in tobacco suspension cell [27]. Peptide fractions isolated from *Arabidopsis* leaves and the synthetic 23-amino acid peptide *At*Pep1 were analyzed using a suspension culture of *Nicotiana tabacum* cells as a universal alkalinization bioassay system. The stress signaling nature of the peptide *At*Pep1 was confirmed in experiments with *Arabidopsis* excised leaves, where it induced defense gene expression and hydrogen peroxide accumulation [20].

Plant regulatory peptides are characterized by their extremely low abundance in cells and tissues, high diversity, and variability throughout the plant’s life cycle [49]. These factors pose significant technical obstacles to the study of plant peptides and hinder the discovery of new peptide regulators [19,49,52]. Complex preparative schemes, typically including reverse-phase (C18) HPLC, are being developed to isolate and purify peptides from plant extracts [6,7,9,20,33]. Peptide bioassays are model experiments where synthetic peptides or peptide fractions are added to cell culture media or applied to plants as foliar sprays to study their effects on plant growth, development, and stress and defense reactions. Bioassay-guided purification was routinely used to validate the biological activity of peptides during preparative HPLC fractionation, enabling the discovery of the first regulatory plant peptides (Figure 1, Figure 2, Figure 3, Figure 4 and Figure 5, Table 1) [6,7,18,20,27]. To facilitate and reduce the number of peptide purification steps, such as tissue disruption and extraction of peptide fractions, special cultures of submerged whole plants [34] and isolated roots [52] overexpressing the protein precursors of peptides have been used. Peptide fractions isolated and purified by HPLC were analyzed using Edman degradation and mass spectrometry to determine their amino acid sequences and identify individual peptides.

After years of research, the structure of the key *Arabidopsis* growth and development regulator CLAVATA3 (CLV3), previously thought to be a small protein, was finally determined to be a short, 12-amino acid peptide using MALDI-TOF-MS/MS [29,30,31]. Mass spectrometry has become an increasingly powerful and indispensable method for identifying endogenous plant peptides since 2006, when the complete amino acid sequences of the dodecapeptides CLV3 [9] and TDIF [18] were identified using MALDI-TOF-MS/MS and LC-MS/MS respectively without the need for Edman degradation. Mass spectrometry has practically replaced the Edman degradation method, which was widely used to determine the amino acid sequences of peptides (Table 1, Figure 2, Figure 3, Figure 4 and Figure 5) [6,7,9,18,20,27]. The structures of the peptides PSK from asparagus, PSY1 from *Arabidopsis*, tobacco peptides HypSys and RALF, and *Arabidopsis* defense peptide *At*Pep1 were elucidated through the combined use of mass spectrometry and Edman degradation [7,18,27] (Table 1, Figure 2, Figure 3, Figure 4 and Figure 5).

The development of high-throughput liquid chromatography coupled to tandem mass spectrometry (LC-MS/MS) technically enabled the identification of endogenous peptide pools composed of hundreds of peptides in plant cells and tissues [50,51]. Instead of only analyzing single peptides of special interest, modern mass spectrometers, like the LC-MS/MS Orbitrap, have increasingly been employed for the systematic investigation of multiple peptides and their ensembles in plant cells and tissues. Peptidomics is the study of endogenous peptides in cells, tissues, and organs [49]. It involves the isolation, identification, and characterization of peptides for the purpose of their qualitative, quantitative, and systems analysis. Despite advances in peptidomics, the functional characterization of a vast majority of newly identified and putative peptides remains a challenge. Developing high-throughput bioassays will facilitate the elucidation of the roles of new peptides in regulating plant growth and defense reactions.

## 3. Identified and Putative Regulatory Peptides in Plants

Regulatory peptides of plants usually consist of 5–20 amino acids, but sometimes up to 50 amino acids. In general, plant regulatory peptides can be subdivided into two major classes: secreted peptides and non-secreted peptides [53]. Secreted peptides such as PSK [7], CLV3 (CLAVATA3) [31], CLE41/44 (CLAVATA3/EMBRYO SURROUNDING REGION-related) and TDIF [33], HypSys, PSY [9], CEP [34], RGF/CLEL/GLV [46,54,55], and RALF [27] are characterized by the presence of the N-terminal signal sequence. The non-secreted peptides such as systemin and *At*Pep1 do not contain this signal sequence at the precursor peptide sequence [6,20,53]. Secreted peptides containing several cysteine residues, such as RALPH, are known as cysteine-rich peptides [27]. Cysteine residues are involved in the formation of intramolecular disulfide bonds, which are critical for the functional activity of the peptides [27].

Three main strategies to find and identify novel plant regulatory peptides (bioassay-guided purification, classical genetics, and bioinformatics) have been used over the past 25 years [3,53].

Bioassay-guided purification combines isolation and HPLC purification of peptides from cells and tissues with bioassay screening of peptides for their biological activity [53]. The first plant regulatory peptides including systemin [6], PSK [7], PSY1 [9], HypSys [18], RALF [27], and TDIF [33] were discovered via this approved method from plant cells and tissues (Table 1, Figure 2, Figure 3 and Figure 5).

Classical genetics studies of *Arabidopsis* shoot meristem activity [29,30] and floral organ abscission [56] led to the discovery of proteinaceous ligands CLV3 and IDA and their receptors CLV1 [41] and HAESA [56] involved in the regulation of developmental processes. A genome-wide search for homologs of CLV3 identified a large family of genes that share homology with *CLV3* and contain a conserved motif at the C-terminus (CLAVATA3/EMBRYO SURROUNDING REGION-related or *CLE*-family) (Figure 1) [29,30]. The amino acid sequence of the mature peptide CLV3 was elucidated using in situ MALDI-TOF-MS/MS, followed by *Arabidopsis* shoot and root bioassays [31].

Bioinformatics is a promising technology to search for novel regulatory peptides, given the progress in genomics and transcriptomics [53]. After isolation and characterization, the amino acid sequence and functions of the first plant regulatory peptides, such as systemin [7] and CLV3 [31], their putative homologs and orthologs were established by aligning the genomic sequences of the precursor proteins. The protein precursors of regulatory peptides are typically encoded by multiple genes and consist of 70 to 120 amino acids. The protein precursors of a particular peptide family differ significantly in their amino acid sequences, with the exception of a conserved domain, which is commonly located in the C-terminal region [34,53]. The families of putative peptides, such as PSK, PSY, RALF, CLE, IDA, CEP, and Pep span across different taxonomic groups of plants. Putative systemin [13,57], PSK [58,59], PSY [60], CLE40 [61,62,63,64,65], PpCLE1–7 [66], IDA [67,68,69,70,71], CEP [72], and Pep [73,74,75] were discovered in different plant families and studied using precursor overexpression, knockouts, and solid-phase peptide synthesis combined with the bioassays.

The conserved motif of the precursor protein corresponds to the amino acid sequence of the mature peptide and plays a critical role in its biological function. Overexpression and knockout of the genes encoding the precursor proteins of putative peptides are widely used to study the role of the putative peptides in plant growth and defense [3,28,29,30]. However, the functional activity of putative peptides as small biomolecules can be definitively demonstrated in model experiments using synthetic peptides with putative amino acid sequences.

The amino acid composition of the putative regulatory peptides generally coincides with that of the isolated and mass spectrometry-verified peptides in terms of the number of amino acid residues. For example, after tomato systemin was identified as an 18-amino acid peptide by mass spectrometry, putative systemins from other nightshade plants, such as potatoes and peppers, were hypothesized to be octadecapeptides as well [14,76,77]; however, their amino acid sequences remain to be determined by mass spectrometry. After the structure of CLV3 was identified as a dodecapeptide (12-amino acid peptide) [31], 25 putative dodecapeptides representing the conserved CLE motif of *Arabidopsis* were synthesized using solid-phase peptide synthesis and screened for bioactivity using bioassays [33]. The amino acid sequences of most CLE peptides, including those of utmost importance, remain to be elucidated by mass spectrometry. A whole-plant submerged culture of *Arabidopsis* overexpressing protein precursors of putative peptides was designed to confirm the synthesis and secretion of peptides into the apoplast. The accumulation of the CLE44 dodecapeptide in the culture medium of submerged plants was confirmed using HPLC fractionation and mass spectrometry [34].

Mass spectrometry-based peptide identification is a crucial criterion for confirming whether a gene family with conserved C-terminal domains identified by in silico searches expresses short regulatory peptides [3]. However, identifying predicted peptides in plant cells and tissues is still a highly challenging task. Although many putative peptides such as IDA and CLE40 from *Arabidopsis* have been the subject of great attention as key regulators of plant development for over two decades, they have not been detected in plant extracts by HPLC and mass spectrometry [53]. The possibility of proteolytic cleavage of precursor proteins and the formation of mature short peptides IDA and CLE40 was demonstrated in an artificial expression system. Short peptides IDA (14 amino acids) [70,78] and CLE40 (12 amino acids) [64] were identified using mass spectrometry after transient expression of corresponding precursor proteins along with subtilisin-like (SBT) proteinases in the leaves of *Nicotiana benthamiana*. Novel approaches for peptide isolation, purification, and mass spectrometry are required to detect the mature CLE40 and IDA in *Arabidopsis*, and other important putative peptides in the extracts of plants.

**Figure 6:**
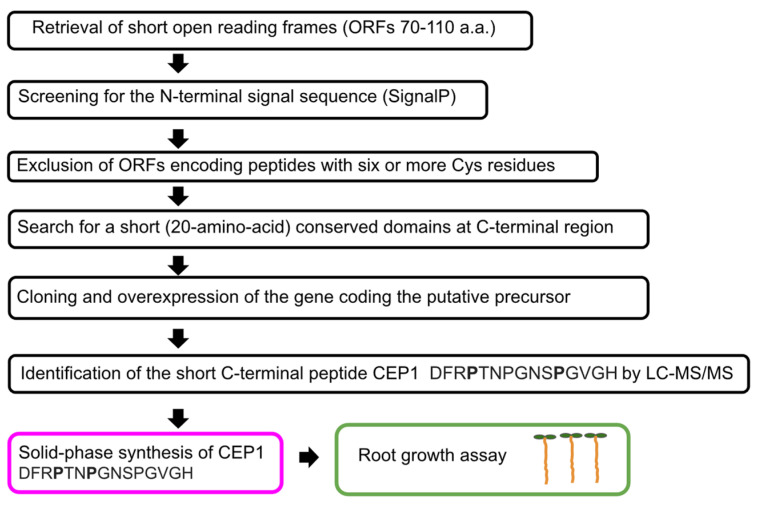
A bioinformatic search algorithm for identifying short bioactive plant peptides, used to discover the regulatory peptide CEP1 [34].

Bioinformatics search algorithms have been developed to identify new putative plant regulatory peptides based on the location of the conserved domain within the C-terminus of small (70–110-amino acid) proteins (Table 1, Figure 6) [34,53]. This approach revealed a putative peptide family CEP encoded by five small *Arabidopsis* genes that share similarity by the conserved 17-amino-acid domain at the C-terminal region [34]. Mass spectrometry analysis of a peptide preparation from the conditioned medium of a whole-plant submerged culture of *Arabidopsis* led to the identification of a 15-amino acid peptide CEP1 from the conserved domain [34]. Bioassays with synthetic CEP1 peptide and overexpression phenotypes demonstrated the root growth regulatory activity of CEP1 [34].

## 4. Bioassays of Synthetic Peptides for Their Biological Activity Are Essential to Elucidate the Functional Role of Isolated and Putative Peptides

Identifying new plant peptides by mass spectrometry and determining their putative homologs using bioinformatics typically involves studying the biological activity and molecular mechanisms of one or several members of the peptide family. The ultimate goal of studying new plant peptides is to identify their receptors and elucidate the molecular mechanisms of their biological activity. The search for peptide receptors in plants is a highly challenging task [79]. To date, receptors have been identified for only a couple of dozen isolated peptides, including tomato systemin [37], PSK [38], IDA [67], CLV3 [41], TDIF/CLE41/44 [42], RALF [43], *At*Pep1 [80], RGF [47], PSY [9,39], CEP1 [45] and CIF [48] (Table 1). Plant peptide receptors have been identified using sophisticated photoaffinity and radioligand methods, but these methods can be time-consuming and labor-intensive. Receptor expression libraries facilitate peptide receptor identification. The CEP, RGF, and CIF peptide receptors were identified using an expression library established in tobacco cultivated cells overexpressing 100 purposefully selected LRR-RLKs from *Arabidopsis* [45,46,48] (Table 1).

While the study of many putative peptides often does not proceed to the identification of their receptors and signaling pathways, bioassaying of synthetic peptides is an essential step in exploring their functional role. New peptides were studied for their biological activity using traditional bioassays and specially designed cell model systems. These bioassays involved exogenous treatment of cells and seedlings with aqueous solutions of peptides at a range from picomolar to micromolar concentrations. Solid-phase synthesis of short peptides with an amino acid composition similar to isolated or putative peptides is a necessary step in bioassay experiments with new plant peptides. Synthetic peptides can be added to cell culture media or applied to plants as foliar sprays in vegetation and field experiments to demonstrate or validate their biological activity (Table 1) [6,31,33].

## 5. Root Growth Assays

After mass spectrometric identification of the isolated dodecapeptides CLV3 from *Arabidopsis* [31] and TDIF from *Zinnia* [33], all the 25 putative *Arabidopsis* CLE dodecapeptides as well as CLV3, which was the 26th member of the CLE peptide family, were synthesized solid-phase and studied using bioassays in model experiments.

The CLV3 dodecapeptide, which is expressed in shoot apical meristems [29], was shown to inhibit meristem cell division in both shoot apical meristems and roots of *Arabidopsis* seedlings [31]. This was demonstrated by treating seedlings with synthetic CLV3 dodecapeptide and analyzing the anatomy of their shoot apical meristems and roots.

Over the past few decades, the traditional method of growing *Arabidopsis* seedlings on Petri dishes in a vertical position has become a standard approach to study a wide range of physiological processes in plants, in particular growth, anatomy, and morphology of roots [81]. Because this bioassay focuses on the root growth and development, rather than the shoots of *Arabidopsis* seedlings, it is called the root growth assay (RGA) [82]. Although many members of the *Arabidopsis* CLE peptide family are not expressed in roots, the corresponding synthetic putative peptides were found to act as root growth inhibitors in RGA [31,33].

Arabidopsis RGA is a sensitive and uncomplicated bioassay for screening new plant peptides [31,33]. It has played a key role in the discovery of such families of plant regulatory peptides as PSY [9], CEP [34], and RGF/CLEL/GLV [46,54,55,83]. Treatment of *Arabidopsis* seedlings with the putative synthetic CLE peptides PpCLE1–7 from the moss *Physcomitrium patens* revealed that the CLE peptide signaling pathway is evolutionarily conserved in land plants [66,84]. In addition to *Arabidopsis* seedlings, other plant species, such as *Medicago truncatula* and rice, can also be used as model plants in RGA. For example, *Medicago truncatula* seedlings have been used to study the effect of the CEP1 peptide on lateral root development [85]. Rice seedlings grown in vertical Petri dishes have been used to bioassay the CLE peptides from *Arabidopsis*. Some *Arabidopsis* CLE peptides inhibited root growth in both *Arabidopsis* and rice, but only reduced the size of the shoot apical meristem in *Arabidopsis* [40]. This demonstrates the versatility of RGA as a bioassay for peptides from different taxonomic groups of plants.

Seedlings grown in vertical Petri dishes are suitable for measuring a variety of root growth and development parameters. In addition to simple and reliable root growth measurements, RGA assays in vertical Petri dishes can also be used to measure the degree of deviation of the main root growth axis from the vertical axis. This parameter is important for studying root gravitropism, and its measurement has been successfully used to discover the GLV regulatory peptide family [55].

The RGA is a suitable system for studying the effects of low-molecular-weight compounds including peptides on growth and differentiation processes [81,85]. A study of the biological activity of the putative moss *Physcomtrium patens* dodecapeptide PpCLE2 using Arabidopsis RGA showed that the synthetic peptide inhibited root growth and stimulated lateral root formation at micromolar concentrations (0.1–1 μM) [84].

Regulatory peptides have a wide range of effective concentrations, from low (micromolar) to ultra-low (picomolar) (Table 1) [6,9,33,72]. The relationship between peptide activity and concentration is often nonlinear, meaning that the effect of the peptide can change in a non-monotonic way [33,84]. Therefore, to study the biological activity of synthetic peptides using bioassays, it is important to test a wide range of concentrations.

## 6. Cellular Bioassays

Cell model systems are widely used to test new bioactive compounds for biotechnology, medicine, and agriculture. Several well-studied plant cell and protoplast cultures have been successful in testing new plant peptides. Suspension and callus cultures of plant cells have played a key role in identifying plant regulatory peptides, such as PSK, PSY1, HypSys, RALF, *At*Pep1, and TDIF (Table 1, Figure 3, Figure 4 and Figure 5) [6,7,9,18,20,27,33].

The family of short regulatory peptides, PSK, was discovered while searching for cytokinetic regulators in liquid medium conditioned during the suspension cultivation of isolated *Asparagus officinalis* L. mesophyll cells (Figure 3). A model asparagus mesophyll cell culture was purposefully developed as a cellular bioassay to search for regulatory peptides and other small biomolecules. In these experiments, cultivated asparagus cells were used as both a source of peptides and a single-celled test system [7]. During the test experiments, the synthetic PSKs were added to the culture medium at micromolar concentrations. As a result, the mitogenic activity of PSKs was confirmed [7]. The regulation of plant cell division mediated by PSKs is also of applied interest for use in crop cultivation. Later, using *Arabidopsis* as a model, it was shown that PSKs are involved in the regulation of the growth of pollen tubes and plant interactions with microbial pathogens [86].

Rice protoplasts are cellular systems that have been used to test the biological activity of PSKs. Rice protoplast bioassays are conducted using agarose blocks with embedded protoplasts in agar medium. The blocks are incubated in liquid medium with or without the test compound. The criterion for phytohormonal activity of the test substance is the intensity of protoplast division and the formation of callus structures in the agar layer, as estimated by counting their number in agarose blocks [87].

The callus culture of *Arabidopsis* was developed as a bioassay system to determine the cytokinetic activity of the sulfated octadecapeptide PSY1, which was isolated from the culture medium of *Arabidopsis* suspension cells (Figure 4). The ability of *Arabidopsis* peptide PSY1 to induce plant cell divisions was confirmed in experiments using *Asparagus officinalis* L. cell culture [9]. Cultivated asparagus cells can be used as a universal cellular bioassay to study the biological activity of new peptides from different taxonomic groups of plants. This demonstrates the versatility of plant cell cultures as bioassay systems for determining the biological activity of peptides from diverse plant families, both isolated and putative, and reveals the technological potential of cultivated plant cells for high-throughput peptide compound screening assays.

The study of conditioning factors that suppress the differentiation of cultivated cells of *Zinnia* into tracheal cells in suspension culture led to the discovery of short peptide regulators of the vascular system of seed plants [33]. It was found that the process of differentiation of cultivated *Zinnia* suspension cells into tracheal elements is inhibited by the dodecapeptide TDIF, which turned out to be a member of the large CLE family of peptide phytohormones. Surprisingly, the amino acid sequence of the dodecapeptide TDIF from *Zinnia* (HEV**P**SG**P**NPISN, Table 1) is identical to that of the CLE41/44 peptides from *Arabiodopsis*. These results made it possible to use the suspension culture of *Zinnia* cells as a universal model system for testing peptide regulators of cell differentiation not only from *Zinnia*, but also from other plant families.

The combination of two different bioassays in the study of the biological activity of synthetic putative CLE dodecapeptides gave an unexpected result.

Most of the tested CLE peptides suppressed the root growth in Arabidopsis RGA and did not affect the processes of cellular differentiation in the experiments with *Zinnia* cells [31]. Two synthetic CLE dodecapeptides (CLE41/44 and CLE42) had no effect on root growth, but they showed activity as negative regulators of differentiation of vascular *Zinnia* cells. Putative CLE peptides were classified into two distinct groups based on their biological activity. Peptides from the first group were classified as A-type peptides because they are active as root growth regulators, but do not affect cell differentiation. The second type of CLE peptides, B-type, do not affect *Arabidopsis* root growth, but are active as a regulator of cell differentiation in *Zinnia* xylogenic cell culture [88]. Thus, two different bioassays, one based on cultured xylogenic *Zinnia* cells and the other based on *Arabidopsis* roots, were complementary in one experiment. Their simultaneous use made it possible to identify two types of regulatory peptides that differ fundamentally in biological activity (growth and differentiation) within the same family of putative *Arabidopsis* CLE peptides.

## 7. Bioassays Based on Cellular Stress Reactions

The development of bioassays for the search and identification of plant signal defense peptides is a complex task, for which it is very difficult to find a universal and high-throughput system. A well-known cellular model system helped overcome the challenge of studying cellular stress response using the electrochemical proton gradient in cultivated plant cells. During the study of the cellular proton gradient, a highly sensitive suspension culture of *Nicotiana tabacum* cells was developed. It is widely used as a bioassay system to detect rapid liquid medium alkalinization induced by stress factors. The development of a bioassay based on the alkalinization of the medium of tomato suspension-cultured cells in response to systemin, which is characterized by an increase in pH (up to 1 unit per 10 min), facilitated the search for peptide signals in tobacco [89]. Focusing on the low-molecular factors affecting pH changes in tobacco cell culture, the pioneers of tomato systemin discovered the tobacco octadecapeptide systemins HypSys [18] (Table 1, Figure 5). In the same series of experiments with a model culture of tobacco cells, by the criterion of increasing the pH of the extracellular culture medium, the Rapid alkalinization factor (RALF) peptide elicitor was discovered, which has a significantly higher molecular weight than HypSys, namely 5 kDa, and two intramolecular cysteine bridges, which were critical for its biological activity [27] (Table 1, Figure 5). Bioinformatics searches have shown that the amino acid sequence of the RALF polypeptide is highly conserved within the plant kingdom, and RALF peptide homologs are found in a wide circle of crop plants such as potatoes, wheat, barley, corn, soybeans, sorghum, and cotton [90]. The RALF polypeptide was obtained by solid-phase synthesis and transferred to a functionally active configuration with two closed cysteine bridges for bioassays [27]. It was found that the addition of RALF to the incubation medium causes inhibition of root growth, consistent with the results of its testing on cell cultures in terms of alkalinization of the cultivation medium. These results suggested that the RALF peptide is involved in the basic and universal mechanisms of growth and development of higher plants [27], which was confirmed in more recent studies of its effect on nodule formation and pollen tube growth [91]. In 2014, the receptor for the RALF peptide, the membrane protein FERONIA (FER), belonging to the Cr-RLK family of receptor-like kinases, was identified [91].

Further search and identification of plant defense signaling peptides using a bioassay based on the measurement of medium alkalinization in tobacco suspension culture helped the pioneers of systemin to isolate and identify the 23-amino acid protective peptide *At*Pep1 from the leaf extract of *Arabidopsis* (Table 1, Figure 5). *At*Pep1 was active in the sub-nanomolar concentration range and caused an increase in hydrogen peroxide production and defensin expression [20]. This study demonstrates that tobacco cell suspension culture can be used as a universal alkalinization cell model for the search and identification of plant defense peptides from other plant families.

The molecular mechanisms of biological activity of *At*Pep1 involve interaction with the leucine-enriched membrane receptor kinase PEPR1 and are under the control of such stress regulators as methyl jasmonate and methyl salicylate [21]. *At*Pep1–8 peptides, as well as their orthologs from a number of cultivated seed plant families, are classified as plant elicitor peptides (Peps) and attract the attention of researchers as promising protective preparations for crop production. It has been established that Peps, as well as their receptors, differ significantly between species of various families of cultivated plants. At the same time, molecular downstream signaling systems from the Pep-PEPR ligand-receptor system show common features among plants from various seed plant families. It is suggested that Peps co-evolved with their PEPR receptor in each individual plant taxonomy group, and the molecular signaling system downstream of PEPR is evolutionarily conserved among plants belonging to different families [92]. Treatment of *Arabidopsis* and maize plants with ultra-low concentrations of Pep peptide water solutions has shown that they can be used as preparations that enhance the systemic defense responses of agricultural plants [21,22].

Potato peptide elicitor *St*Pep1 is of significant applied interest as a signal inducer of potato defense reactions. In model experiments, it was found that the treatment of potato plants with *St*Pep1 peptide elicitor at low concentrations (100 nM–1 μM) leads to an increase in resistance to nematodes, when plants of the Russet Burbank variety grown *in vitro* are artificially infected with the phytopathogenic *Meloidogyne chitwoodi* [93]. Suspension-cultured potato cells are highly sensitive to molecular stress factors and alkalinize the culture medium in response to their action, similar to that observed in tobacco cultivated cells [94]. The alkalinization bioassay based on plant suspension cell cultures is proposed as a marker system to study the early stages of plant defense reactions [95]. This highly sensitive cellular approach can be used to screen newly identified and putative signaling peptides for their signaling potential during stress and defense reactions.

## 8. Conclusions

Dozens of plant regulatory peptides have been identified in the past two decades using HPLC fractionation and mass spectrometry. These peptides have been shown to play a key role in the regulation of plant growth, development, defense, and stress resistance. Aqueous solutions of synthetic regulatory peptides can be used as the environmentally friendly biostimulants to improve plant growth and protection due to their physiological effects at low and ultra-low concentrations. Hundreds of putative regulatory peptides have been predicted by identifying families of precursor proteins with conserved motifs at the C-terminus, using both sequence alignments of isolated peptides and bioinformatics search algorithms. Synthetic putative peptides are promising bioregulators for crop production and defense, even though their presence in plant cells and tissues has not been directly confirmed [96,97]. Putative peptides are believed to have regulatory functions similar to their isolated homologs. The hypothetical functions of putative plant peptides provide a rational basis for further study of their biological activity. The solid-phase synthesis of peptides and their screening using universal bioassays are essential steps in elucidating molecular mechanisms of isolated and putative plant peptides. A single bioassay is not sufficient to identify regulatory peptides involved in a specific physiological process [49]. Simultaneous peptide screening using different bioassays can reveal peptides within a single peptide family that have different physiological effects [9]. Bioassays of synthetic plant peptides at a wide range of concentrations can significantly improve the identification and validation of their biological activity. The development of high-throughput screening systems for short peptides is critical for the fields of plant biochemistry and agricultural biotechnology.

## Figures and Tables

**Table 1:** Isolation and characterization of plant regulatory peptides.

Peptide, Reference Amino Acid Sequence, Plant Species	Methods of Isolation and Identification	Bioassay/Plant Species	Effective Concentration	Biological Activity	Receptor, Reference
Systemin [6] AVQSK**PP**SKRD**PP**KMQTD *Solanum lycopersicum*	Bioassay-guided purification, Edman degradation	Proteinase inhibitor quantification in plant cuttings of *Solanum lycopersicum*	40 pM	Regulation of systemic response to herbivore attack	SYR1 [36,37]
PSK (Phytosulfokine) [9] **Y**I**Y**TQ *Asparagus officinalis*	Bioassay-guided purification, Edman degradation, LC-MS/MS	Cellular mitogenic bioassay based on the suspension of cultivated cells of *Asparagus officinalis*	1 nM–1 μM	Regulation of cell division and expansion	PSKR1 [38] PSKR2 [9]
PSY1 (Plant peptide containing sulfated tyrosine 1) [9] D**Y**GDPSANPKHDPGV**PP**S *Arabidopsis thaliana*	Bioassay-guided purification, Edman degradation, LC-MS/MS	Cellular mitogenic bioassay based on the suspension of cultivated cells of *Asparagus officinalis* Root growth bioassay based on *Arabidopsis thaliana* seedlings	1 nM–0.1 μM	Regulation of cell division and expansion	PSY1R [39]
CLV3 (CLAVATA3) [31,40] RTV**P**SG**P**DPLHH *Arabidopsis thaliana*	Overexpression of precursor, MALDI-TOF—MS/MS	Root growth bioassay based on *Arabidopsis thaliana* and *Oryza sativa* seedlings Cellular differentiation bioassay based on the suspension of cultivated cells of *Zinnia elegans*	1 μM	Regulation of cell proliferation in the meristem	CLV1 [41]
TDIF (Tracheary element differentiation inhibitory factor) [31] HEV**P**SG**P**NPISN *Zinnia elegans*	Bioassay-guided purification, Edman degradation, LC-MS/MS	Cellular differentiation bioassay based on the suspension of cultivated cells of *Zinnia elegans* Root growth bioassay based on *Arabidopsis thaliana* seedlings	10 pM–8 nM	Regulation of differentiation of vascular cells	PXY [42]
HypSys (Hydroxyproline-rich systemins) [18] RGANLP**PP**S**P**ASS**PP**SKE *Nicotiana tabacum*	Bioassay-guided purification, Edman degradation, LC-MS/MS	Alkalinization cellular bioassay based on suspension cultivated cells of *Nicotiana tabacum*	0.2 nM–2 nM	Regulation of stress and defense reactions	n.d.
RALF * (Rapid alkalinization factor) [27] ATKKYISYGALQKNSVPCSRRGASY-YNCKPGAQANPYSRGCSAITRCRS *Nicotiana tabacum*	Bioassay-guided purification, Edman degradation, LC-MS/MS	Alkalinization cellular bioassay based on suspension cultivated cells of *Nicotiana tabacum* and *Solanum lycopersicum* Root growth bioassay based on *Arabidopsis thaliana* and *Solanum lycopersicum* seedlings	2–10 nM (Alkalinization bioassay) 1 μM (Root growth bioassay)	Regulation of growth, development and stress reactions	FER [43]
*At*Pep1 (plant elicitor peptide) [20,22] ATKVKAKQRGKEKVSSGRPGQHN *Arabidopsis thaliana*	Bioassay-guided purification, Edman degradation, LC-MS/MS	Alkalinization cellular bioassay based on suspension of cultivated cells of *Nicotiana tabacum*	10 nM	Activating signaling pathways associated with stress and defense responses	PEPR1 [44]
CLE44 (CLAVATA3/EMBRYO SURROUNDING REGION-related 44) [34] HEV**P**SG**P**NPISN *Arabidopsis thaliana*	In silico screening and overexpression of precursor, LC-MS/MS	Cellular differentiation bioassay based on the suspension of cultivated cells of *Zinnia elegans* Root growth bioassay based on *Arabidopsis thaliana* seedlings	10 pM–8 nM	Regulation of differentiation of vascular cells	PXY [42]
CEP1 (C-terminally encoded peptide) [34] DFR**P**TNPGNS**P**GVGH *Arabidopsis thaliana*	In silico screening and overexpression of precursor LC-MS/MS	Root growth bioassay based on *Arabidopsis thaliana* seedlings	1 μM	Root growth and development	CEPR1 [45] CEPR2 [45]
RGF1 (Root meristem growth factor 1) [46] D**Y**SNPPGHHP**P**RHN *Arabidopsis thaliana*	In silico screening and overexpression of precursor LC-MS/MS	Root growth bioassay based on *Arabidopsis thaliana* seedlings	100 pM–100 nM	Root growth and development	RGRF1 RGFR2 RGFR3 [47]
CIF1 (Casparian strip integrity factor 1) [48] DYGNNS**P**S**P**RLERPPFKLIPN *Arabidopsis thaliana*	In silico screening and overexpression of precursor LC-MS/MS	Root growth bioassay based on *Arabidopsis thaliana* seedlings	100 nM	Promoting lignin deposition and reinforcing the structural integrity of the endodermal layer	GSO1/ SGN3 [48]

* The RALPH peptide’s 49-amino acid sequence spans two lines. Bold in peptide sequences indicates posttranslational modifications: proline hydroxylation and tyrosine sulfation.

## Abbreviations

List of abbreviations	
HPLC	High-performance liquid chromatography is an analytical technique for the separation and quantification of chemical compounds
LC-MS/MS	Liquid chromatography—tandem mass spectrometry
LRR	Leucine-rich repeat
MALDI	Mass spectrometry technique matrix-assisted laser desorption/ionization
MALDI-TOF-MS	Matrix-assisted laser desorption/ionization time-of-flight mass spectrometry
MALDI-TOF-MS/MS	Matrix-assisted laser desorption/ionization time-of-flight tandem mass spectrometry
MAPK	Mitogen-activated protein kinases
RGA	Root growth assay
RTK	Receptor tyrosine kinase
RLK	Receptor-like kinase
SBT	Subtilisin-like serine proteinase
WT	Wild type
List of peptide acronyms	
*At*Pep1	The 23-amino acid peptide isolated from extracts of *Arabidopsis* leaves, exhibiting characteristics of an endogenous elicitor of the innate immune response [20], the first member of the Pep (plant elicitor peptides) family [22]
CEP	C-terminally encoded peptide [34]
CIF	Casparian strip integrity factor 1 [48]
CLE	CLAVATA3/EMBRYO SURROUNDING REGION-related [30]
CLEL	CLE-like peptide [54]
GLV	GOLVEN peptide [55]
HypSys	Hydroxyproline-rich peptide systemin [18,77]
Pep	Plant elicitor peptide [22,23]
RALF	Rapid alkalinization factor [27]
RGF	Root meristem growth factor peptide [46]
PSY	Plant peptide containing sulfated tyrosine [9]
TDIF	Tracheary element differentiation inhibitory factor peptides [33]
